# Environmental and energetic exergetic sustainability of stevia leaf drying in a PVT indirect solar dryer with variable airflow and tray levels

**DOI:** 10.1038/s41598-025-24654-9

**Published:** 2026-01-28

**Authors:** Abdallah Elshawadfy Elwakeel, Awad Ali Tayoush Oraiath, Abubakr Tantawy, András Székács, Omar Saeed, Mohamed Hamdy Eid, Samy F. Mahmoud, Huda Aljumayi, Rahmah N. AlQthanin, Said Elshahat Abdallah, Ibraheem A. H. Yousif, Aml Abubakr Tantawy

**Affiliations:** 1 Agricultural Engineering Department, Faculty of Agriculture and Natural Resources, Aswan University, Aswan, 81528 Egypt; 2 Department of Agricultural Engineering, Faculty of Agriculture, Omar Al Mukhtar University, P.O. Box 991, Al Bayda, Libya; 3 Agronomy Department, Faculty of Agriculture, Minia University, Minia, Egypt; 4 Agro-Environmental Research Centre, Institute of Environmental Sciences, Hungarian University of Agriculture and Life Sciences, Páter, Károly U. 1, Gödöllő, 2100 Hungary; 5 Doctoral School of Environmental Science, Hungarian University of Agriculture and Life Sciences (MATE), Páter Károly U. 1, Gödöllő, 2100 Hungary; 6 Institute of Environmental Management, Faculty of Earth Science, University of Miskolc, Miskolc- Egyetemváros, 3515 Hungary; 7 Geology Department, Faculty of Science, Beni-Suef University, Beni-Suef, 65211 Egypt; 8 Department of Biotechnology, College of Science, Taif University, Taif City, Saudi Arabia; 9 Department of Food Science and Nutrition, College of Science, Taif University, 21944 Taif, Saudi Arabia; 10 Department of Biology, College of Science, King Khalid University, 61431 Abha, Saudi Arabia; 11 Prince Sultan Bin Abdulaziz for Environmental Research and Natural Resources Sustainability Center, King Khalid University, 61421 Abha, Saudi Arabia; 12 Agricultural Engineering Department, Faculty of Agriculture, Kafrelsheikh University, Kafrelsheikh, Egypt; 13 Soil and Water Science Department, Faculty of Agriculture, Cairo University, Giza, 12613 Egypt; 14 Food Science Department, Faculty of Agriculture, Beni-Suef University, Beni-Suef, 65211 Egypt

**Keywords:** Sustainability indicators, Environmental indicators, Solar drying, Renewable energy, Solar energy, Energy science and technology, Engineering, Environmental sciences

## Abstract

During the current study, *Stevia rebaudiana* leaves were dried using a photovoltaic thermal–indirect solar dryer (PVT-ISD), and the influence of tray position inside the drying chamber was assessed using six trays under two airflow rates (0.08 and 0.13 m^3^/s) with a uniform layer thickness of 3 cm. The fastest drying occurred on the lowest tray, closest to the hot air inlet, particularly at the higher airflow rate, achieving a final moisture content of 5.7%. Energy analysis showed that thermal efficiency of the solar collector reached up to 55.7% at the higher airflow. Exergy analysis supported this improvement, with maximum exergy efficiency of the solar collector increasing to 14.68%. Conversely, the drying room performed better at the lower airflow, reaching a maximum exergy efficiency of 37.89%. Sustainability indicators revealed slight improvements in the solar collector but declines in the drying room at higher airflow. Environmental indicators further demonstrated enhanced performance of the solar collector at higher airflow, with maximum reductions observed in the environmental destruction coefficient (19.6%), environmental impact factor (20.6%), and environmental effect factor (20.4%).

## Introduction

Stevia (*Stevia rebaudiana Bertoni*) is a perennial shrub native to South America, renowned for its intensely sweet leaves, which have been used for centuries as a natural sweetener and traditional medicine. The plant’s sweetness comes from steviol glycosides—compounds that are 100 to 300 times sweeter than sucrose but contain no calories, making stevia an attractive alternative to sugar for those seeking to reduce caloric intake or manage conditions like diabetes and obesity^1–4^.

Drying stevia leaves is crucial for preserving their quality, extending shelf life, and maintaining the concentration of valuable compounds like steviol glycosides and antioxidants. Various drying methods—including hot air, infrared, vacuum, solar, microwave, and shade drying—significantly affect the leaves’ moisture content, nutrient retention, color, and microstructure^5–7^. Overall, the drying method and conditions must be carefully selected to balance efficiency, energy use, and the preservation of stevia’s desirable qualities ^8–12^.

Mechanical dryers offer a practical alternative to traditional open-air drying (OAD) methods, especially for agricultural produce, by addressing many of the limitations associated with these conventional techniques^13–16^. These drying systems are generally categorized based on their energy input—either as conventional (fossil fuel-based) or non-conventional (renewable energy-based). Conventional systems rely heavily on fossil fuels, which not only raise operational costs but also contribute significantly to environmental pollution. In contrast, non-conventional dryers, such as those powered by solar energy, present an eco-friendly and cost-effective solution that reduces both energy use and environmental harm^17–22^. By employing solar energy in enclosed drying systems, producers can avoid the typical drawbacks of open-air drying like oil, gas, and coal, thereby cutting down greenhouse gas emissions and other pollutants^23–27^. Solar drying is widely recognized as a sustainable and efficient method of food preservation, owing to its effective use of abundant solar radiation^28^. Compared to traditional OAD methods, solar dryers reduce drying time, limit post-harvest losses, and improve product quality^29–35^. The application of solar drying in regions like Aswan, Egypt, is particularly promising. Aswan recorded the highest global temperatures in 2024, offering optimal climatic conditions for solar drying operations. The region’s intense solar radiation and high ambient temperatures make it well-suited for moisture removal from crops while maintaining their quality. As a result, local farmers are increasingly shifting toward solar-based drying systems to enhance efficiency and minimize reliance on outdated practices. Additionally, Egypt benefits from a high average daily solar radiation (about 8 kWh/m^2^/day) and nearly 11 h of sunshine per day, making it a prime location for solar energy applications in agriculture^36–38^. Overall, previous research underscores the intricate relationship between drying variables—such as temperature, airflow velocity, radiation intensity, and energy input—and their collective impact on drying efficiency, product quality, and the biochemical integrity of stevia leaves. Recent advancements, including hybrid solar drying systems^5,39–41^, far-infrared radiation^7,42^, freeze drying^6^, and microwave-assisted techniques^43^, have further enhanced the potential for effective and nutrient-retentive drying.

The performance evaluation of solar dryers cannot rely solely on a single assessment method, as each approach highlights different aspects of system behavior. Traditional energy analysis (first law) provides useful information about energy utilization and thermal efficiency, yet it considers only the quantity of energy and neglects the quality or degradation of energy during the drying process^44–49^. Consequently, a dryer may appear energetically efficient while still suffering from significant losses caused by irreversibilities. To address this limitation, exergy analysis (second law) is employed, as it accounts for both the quantity and quality of energy. Exergy analysis not only quantifies the useful portion of energy but also identifies where and how inefficiencies occur within the system components, thereby offering deeper insights into possible design and operational improvements^44,45,50^. However, high energy and exergy efficiencies alone do not necessarily guarantee long-term sustainability^51–53^. Solar dryers must also be evaluated in terms of their environmental and resource-related impacts. Sustainability indicators, such as the sustainability index, waste exergy ratio, and environmental destruction coefficient, extend the evaluation framework to include ecological responsibility and resource conservation^45^. Integrating energetic, exergetic, and sustainability indicators therefore provides a comprehensive perspective that combines technical performance, thermodynamic effectiveness, and environmental viability. This holistic assessment is essential for optimizing solar dryer design, improving energy use, reducing environmental impact, and ensuring alignment with global sustainability goals^46,50,51^.

Despite notable advancements in solar drying technologies, limited research has addressed the combined influence of tray position and airflow rate on drying performance and the environmental implications of stevia drying. These parameters are particularly critical in multi-tray drying systems, where uneven airflow distribution across tray levels often leads to inconsistent drying rates, non-uniform moisture content, and potential quality deterioration. To address this gap, the present study systematically investigates the effects of tray position and variable airflow rates on the drying kinetics and environmental performance of a PVT-indirect solar dryer applied to stevia leaves. The findings are expected to generate novel insights that can inform the optimization of dryer design, enhance operational efficiency, and improve product quality, thereby contributing to more sustainable stevia processing.

## Materials and methods

### Description of the PVT-ISD

**Fig. 1:**
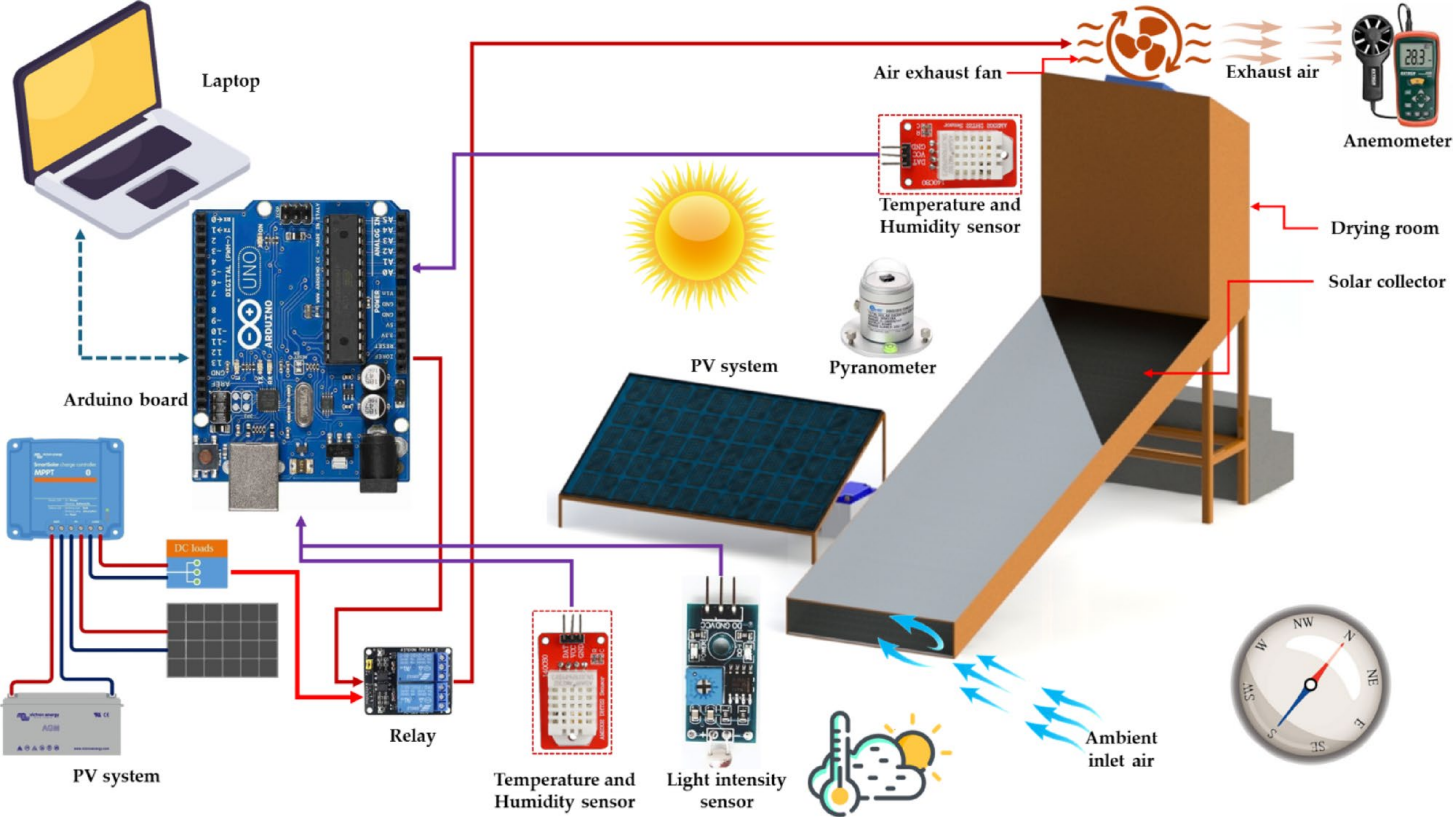
Operating map of the PVT-ISD.

A well-insulated drying chamber was constructed using durable, heat-resistant materials (glass wool) to minimize thermal losses during operation. It was designed as a multi-tier structure and fitted with six evenly spaced perforated trays to support uniform layers of stevia leaves (20 cm interval). These trays were arranged vertically to enable the investigation of drying performance at various heights within the chamber. In this study, the drying chamber was specifically utilized to examine the effects of tray position on drying kinetics, moisture removal efficiency, and final product quality. The illustrated system in Fig. 1 represents an IoT-integrated photovoltaic-thermal (PVT) indirect solar dryer (ISD) (PVT-ISD) designed for efficient and smart drying of agricultural products. It utilizes a PV panel to generate electrical energy and a thermal collector to heat air entering the drying chamber. The system is equipped with a DHT22 (AM2302) temperature and relative humidity sensor (manufactured by Aosong Electronics Co., Ltd., China), positioned inside the drying chamber to monitor environmental conditions. Solar radiation is measured using a pyranometer (SENTEC RS485, Sichuan), while airflow rate at the exhaust is monitored with a digital anemometer (Extech AN100, China). All sensor data are collected and processed by an Arduino Uno R3 microcontroller (developed by Arduino.cc, Italy), which also controls the 12 V DC exhaust fan to regulate air circulation. A microSD card module is integrated as a data logger to store real-time sensor readings. The entire system is powered by a PV system (320W), charge controller, and connected to a 12 V DC battery for energy storage.

### Experimental procedure

#### Sample preparation

**Fig. 2:**
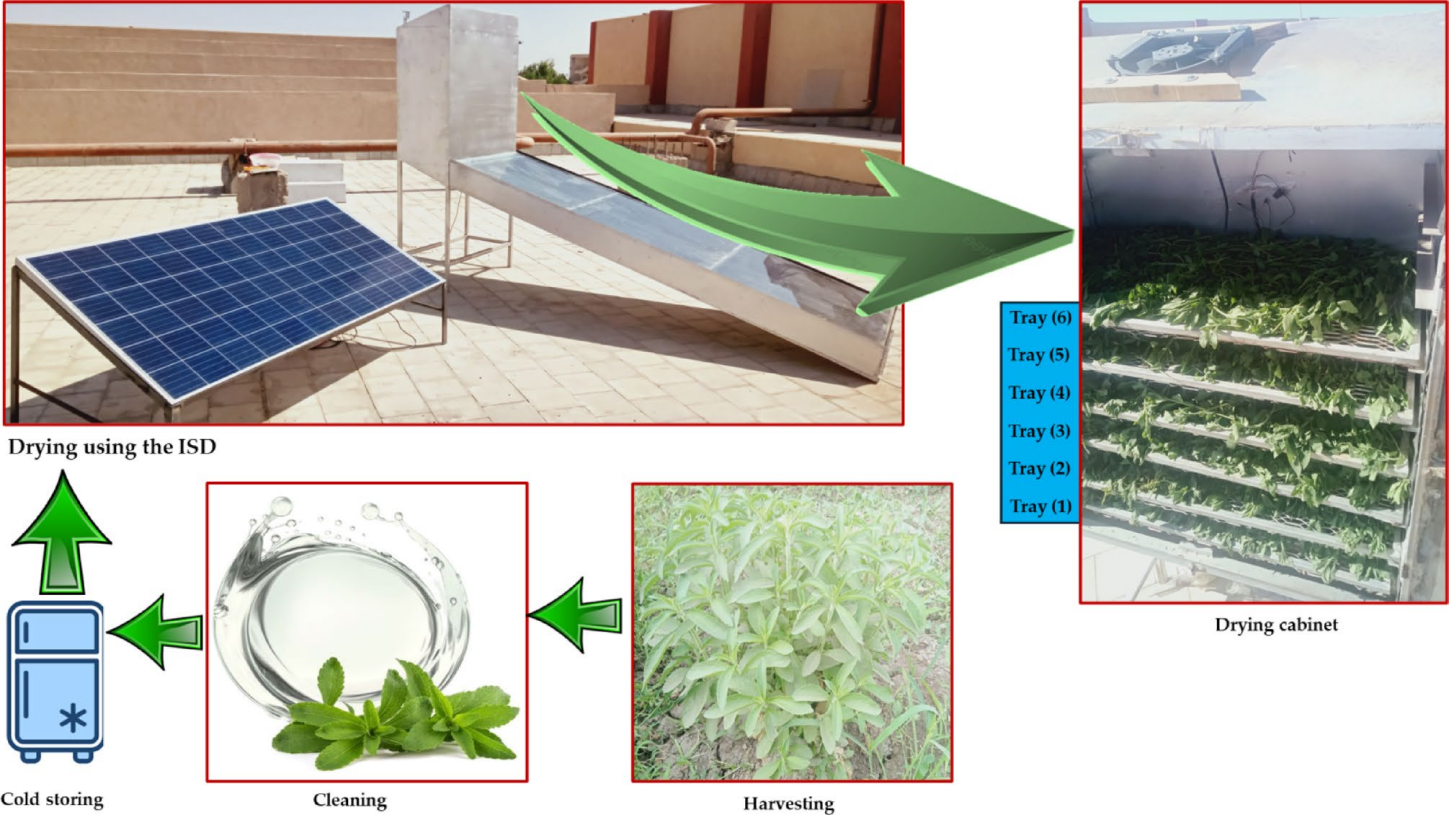
Diagrammatic representation of the stevia sample preparation and drying workflow in the PVT-ISD system.

Fresh *Stevia rebaudiana* leaves were harvested from the experimental farm at Minia University and transported to Aswan University in May 2025 under controlled handling conditions to preserve quality prior to processing. Upon arrival, the leaves were thoroughly cleaned to remove dust, soil particles, and extraneous materials. The cleaned leaves were then temporarily stored in clean, perforated plastic bags to maintain ventilation, reduce condensation, and minimize pre-drying moisture loss (Fig. 2). The average initial moisture content of the leaves was approximately 78.5% (w.b.), determined prior to drying.

#### Loading arrangement in the drying chamber

The drying chamber had a total loading capacity of 4500 g, which was evenly distributed across six trays, with each tray containing 750 g of stevia leaves. For the experiments, the leaves were carefully and uniformly spread in a 3 cm layer thickness across the trays to ensure consistent exposure to airflow and standardized drying conditions (Fig. 2).

The trays were vertically arranged inside the solar drying chamber with approximately 20 cm spacing between consecutive levels. This configuration facilitated unobstructed airflow and promoted efficient and uniform moisture removal across all tray positions.

#### Measurement of moisture loss

The mass change of the stevia samples was systematically monitored at 1-h intervals over the entire drying period. Measurements were performed using a precision electronic balance with an accuracy of ± 0.01 g, ensuring reliable tracking of moisture removal. At each interval, trays were carefully withdrawn, weighed, and immediately returned to the drying chamber to minimize disruption of the drying environment and airflow conditions.

### Performance analysis

#### Drying kinetics

The freshly harvested Stevia rebaudiana leaves were subjected to oven drying at a controlled temperature of 50 °C using a laboratory-grade electric convection oven. Drying continued until the samples attained a constant weight, indicating that all free moisture had been effectively removed. This standard gravimetric method ensured accuracy in determining the true moisture content. Subsequently, the initial moisture content (MC) of the fresh stevia leaves was calculated based on weight loss using Eq. (1), which expresses moisture content on a wet basis^54^.

1 $$MC \left( {w.b.} \right) = \left[ {\frac{{W_{w} - W_{d} }}{{W_{w} }}} \right] \times 100$$

where ${W}_{w}$ and ${W}_{d}$ is the wet and dry weight of stevia sample, respectively in g.

The drying rate (DR) of the stevia leaf samples under varying experimental conditions was determined using Eq. (2), as outlined by standard drying kinetics methodology^54^. This calculation reflects the rate at which moisture was removed from the product over time, providing critical insights into the efficiency of the drying process. By analyzing the drying rate, variations in drying behavior across different tray positions and airflow rates could be quantitatively assessed, thereby contributing to a better understanding of moisture migration dynamics and overall dryer performance.

2 $$\left( {g_{water} /g_{dry\;matter} .h} \right) = \frac{Weight\;loss \left( g \right)}{{\Delta t \left( h \right)}}$$

#### Energy analysis

The PVT-ISD consists primarily of two key units: the solar collector (SC) and the drying room (DR). The performance of these components was evaluated using the fundamental thermodynamic principles of mass and energy conservation under steady-flow conditions, as described by Eqs. (3–10) as stated by Mugi and Chandramohan^55^, and Ekka and Muthukumar^56^. According to these principles, the mass flow rate of air remains constant throughout the system, implying that the amount of air entering the system at the inlet is exactly equal to the amount left at the outlet. This assumption of steady-state operation ensures reliable analysis of heat and mass transfer processes within the dryer and is critical for accurately assessing thermal performance and energy efficiency.

3 $$\sum {\dot{m}}_{ai}=\sum {\dot{m}}_{ao}$$

where ${\dot{m}}_{ai} and {\dot{m}}_{ao}$ are the inlet and outlet mass flow rate of air, respectively.

According to energy conservation principles, the rate of energy transfer by work done ($\dot{W}$), heat ($\dot{Q}$) and mass (${\dot{m}}_{ai}$) into the PVT-ISD, equal to the rate of energy transfer by work, heat and mass coming out of the PVT-ISD (${\dot{m}}_{ao}$), according to Eqs. 4 and 5^57–59^.

4 $$\sum {\dot{E}}_{ai}=\sum {\dot{E}}_{out}$$

5 $$\dot{Q}+\sum {\dot{m}}_{ai}\left({h}_{ai}+\frac{{v}_{ai}^{2}}{2}+{z}_{i}g\right)=\sum {\dot{m}}_{ao}\left({h}_{ao}+\frac{{v}_{ao}^{2}}{2}+{z}_{o}g\right)+\dot{W}$$

where ${h}_{ai} and {h}_{ao}$ are the input and output enthalpy of air, ${v}_{ai} and {v}_{ao}$ are the input and output air velocity, and ${z}_{i} and {z}_{o}$ represent input and output height of datum air. According to the PVT-ISD, the work done ($\dot{W}$) equal zero, and the value of $\left(\frac{{v}_{ai}^{2}}{2}-\frac{{v}_{ao}^{2}}{2}\right)$ is very small and can be neglected.

Equations 6 and 7 were obtained for the SC form Eqs. 3 and 4.

6 $$\sum {\dot{m}}_{ai}=\sum {\dot{m}}_{ao}=\sum {\dot{m}}_{a}$$

7 $$\dot{Q}={\dot{Q}}_{u, \mathrm{SC}}= {\dot{Q}}_{in, \mathrm{SC}}-{\dot{Q}}_{ls, \mathrm{SC}}={\dot{m}}_{a}\left({h}_{ao}-{h}_{ai}\right)$$

where the ${\dot{Q}}_{in, \mathrm{SC}}$, ${\dot{Q}}_{u, \mathrm{SC}}$ and ${\eta }_{en, SC}$ of the SC were calculated according to Eqs. (8–10)^57–59^.

8 $${\dot{Q}}_{in, \mathrm{SC}}={I}_{s}{A}_{SC}$$

where ${I}_{s}$ is solar radiation flux in W/m^2^ and ${A}_{SC}$ is the area of the SC in m^2^ (equal 3 m^2^).

9 $${\dot{Q}}_{u, \mathrm{SC}}={\dot{m}}_{a}{C}_{pa}\left({T}_{co}-{T}_{ci}\right)$$

where ${C}_{pa}$ is the specific heat of air in kJ/kg K, ${T}_{co}$ and ${T}_{ci}$ are the temperatures of air at SC outlet and inlet.

10 $${\eta }_{en, SC}=\frac{{\dot{Q}}_{u, \mathrm{SC}}}{{\dot{Q}}_{in, \mathrm{SC}}}=\frac{{\dot{m}}_{a}{C}_{pa}\left({T}_{co}-{T}_{ci}\right)}{{I}_{s}{A}_{SC}}$$

#### Exergy analysis

Exergy represents the available or usable energy within the PVT-ISD and serves as an indicator of the quality of energy in the system. The assessment of exergy in the PVT-ISD is based on the principles of the second law of thermodynamics, which quantifies the potential for energy conversion into useful work. The value of exergy is calculated using Eq. 11^55,56^, providing a comprehensive understanding of how efficiently energy is utilized and how much of it is available for productive use within the system.

11 $$\begin{gathered} Ex = \left( {u - u_{\infty } } \right) - T_{0} \left( {s - s_{\infty } } \right) + P_{0} \left( {v - v_{\infty } } \right) + \frac{{V^{2} }}{2} + g\left( {z - z_{\infty } } \right) \hfill \\ \quad \quad \quad + \mathop \sum \limits_{ch} \left( {\mu_{ch} - \mu_{\infty } } \right)N_{ch} + \sigma A_{i} F_{i} \left( {3T^{4} - T_{\infty }^{4} - 4T_{\infty } T^{3} } \right) \hfill \\ \end{gathered}$$

Equation 12 is a simplified form of Eq. 11, obtained by eliminating variables and terms that have minimal or no significance in the context of the drying process^55,56^. This refinement resulted in a more streamlined and application-specific expression, making it better suited for practical analysis within the drying system framework^55^.

12 $$\dot{Ex}= {\dot{m}}_{a}{C}_{pa}\left(\left(T-{T}_{0}\right)-{T}_{0}ln\left(\frac{T}{{T}_{0}}\right)\right)$$

where ${T}_{0}$ is atmospheric temperature.

##### Exergy analysis of the SC

$\dot{Ex}$ Balance for the SC is given by Eqs. (13–18)^59–61^,

13 $$\sum {\dot{Ex}}_{in, SC}-\sum {\dot{Ex}}_{out, SC}=\sum {\dot{Ex}}_{ls, SC}$$

where ${\dot{Ex}}_{in, SC}$ is the exergy inflow, ${\dot{Ex}}_{out, SC}$ is the exergy outflow and ${\dot{Ex}}_{ls, SC}$ is the exergy loss.

The exergy inflow for SC (${\dot{Ex}}_{in, SC}$), associated with solar radiation falling on surface of the SC, and can be expressed as shown in Eq. (14), as mentioned by Bait^60^.

14 $${\dot{Ex}}_{in, SC}=\left[1-\frac{{T}_{0}}{{T}_{s}}\right]{\dot{Q}}_{in, abs}$$

where ${T}_{s}$ is the sun temperature (6000 K) and ${\dot{Q}}_{in, abs}$ is the solar energy falling on plate in W, and it was calculated according to Eq. (15)^45^,

15 $${\dot{Q}}_{in, abs}=\alpha \tau {I}_{s}{A}_{SC}$$

where $\alpha$ $and \tau$ are the absorptivity (0.95) and transmissivity of glass cover (0.88), respectively^61^.

Exergy outflow for the SC (${\dot{Ex}}_{out, SC}$), refers to the usable energy output available after accounting for losses and irreversibility. It represents the maximum work potential of the thermal energy transferred by the SC to the system, such as for heating of air. Higher exergy outflow indicates better efficiency and effective utilization of solar energy. The (${\dot{Ex}}_{out, SC}$) was estimated using Eq. (16)^59^.

16 $${\dot{Ex}}_{out, SC}={\dot{m}}_{a}{C}_{pa}\left(\left({T}_{co}-{T}_{ci}\right)-{T}_{0}ln\left(\frac{{T}_{co}}{{T}_{ci}}\right)\right)$$

The exergy loss for SC (${\dot{Ex}}_{ls, SC}$) can be evaluated from irreversibility and it is given by Eq. (17), as reported by Akpinar^62^.

17 $$\begin{gathered} \dot{E}x_{ls, SC} = I = T_{0} S_{gen} = \left[ {1 - \frac{{T_{0} }}{{T_{s} }}} \right]\dot{Q}_{in, SC} \hfill \\ \quad \quad \quad \quad \quad - \dot{m}_{a} C_{pa} \left( {\left( {T_{co} - T_{ci} } \right) - T_{0} \ln \left( {\frac{{T_{co} }}{{T_{ci} }}} \right)} \right) \hfill \\ \end{gathered}$$

The exergy efficiency of SC is obtained using Eq. (18)^59,63^.

18 $${\eta }_{ex, SC}=\frac{{\dot{Ex}}_{out, SC}}{{\dot{Ex}}_{in, SC}}=1-\frac{{\dot{Ex}}_{ls, SC}}{{\dot{Ex}}_{in, SC}}=1-\frac{{T}_{0}{S}_{gen}}{\left[1-\frac{{T}_{0}}{{T}_{s}}\right]{\dot{Q}}_{in, \mathrm{SC}}}$$

##### Exergy analysis of the DR

The exergy balance for the DR is established to comprehensively account for all forms of exergy input, output, and associated losses occurring within the system. The balance is mathematically expressed as follows^55,56^:

19 $${\dot{Ex}}_{ls, DR}={\dot{Ex}}_{in, DR}-{\dot{Ex}}_{out,DR}$$

20 $${\dot{Ex}}_{in, DR}={\dot{m}}_{a}{C}_{pa}\left(\left({T}_{in,DR}-{T}_{0}\right)-{T}_{0}ln\left(\frac{{T}_{in,DR}}{{T}_{0}}\right)\right)$$

21 $${\dot{Ex}}_{out, DR}={\dot{m}}_{a}{C}_{pa}\left(\left({T}_{out,DR}-{T}_{ci}\right)-{T}_{0}ln\left(\frac{{T}_{out,DR}}{{T}_{0}}\right)\right)$$

22 $${\eta }_{ex, DR}=\frac{{\dot{Ex}}_{out, DR}}{{\dot{Ex}}_{in, DR}}$$

#### Sustainability indicators

In this study, the performance of the PVT-ISD was assessed through several key exergy-based sustainability indicators. These indicators offer a thorough evaluation of the system’s thermodynamic efficiency by measuring how effectively the input exergy is utilized in relation to the exergy losses^55^.

##### Improved potential (IP)

IP is an exergy-based sustainability indicator that reflects the potential for improving a system’s performance by minimizing exergy destruction. A higher IP value suggests greater room for enhancing the efficiency of the solar drying process^55^.

23 $$IP=\left(1-{\eta }_{ex}\right){\dot{Ex}}_{ls}$$

##### Waste exergy ratio (WER)

The WER quantifies the proportion of input exergy that is not effectively utilized and is lost as waste. It highlights inefficiencies in the drying system, with a higher WER indicating poorer exergy utilization^55^.

24 $$WER=\frac{{\dot{Ex}}_{ls}}{{\dot{Ex}}_{in}}$$

##### Sustainability index (SI)

The Sustainability Index is the ratio of exergy output to exergy destruction and serves as a measure of how sustainably a solar dryer operates. A higher SI indicates better thermodynamic performance and lower irreversibility^55^.

25 $$SI= \frac{1}{1-{\eta }_{ex}}$$

#### Environmental indicators

##### Environmental destruction coefficient (EDC)

EDC evaluates the environmental impact associated with exergy destruction during the drying process. It links thermodynamic inefficiencies to environmental degradation, making it a valuable indicator for green technology assessments^64^.

26 $$EDC= \frac{1}{{\eta }_{ex}}$$

##### Environmental Impact Factor (EIF)

The Environmental Impact Factor assesses the extent to which the operation of a solar dryer contributes to environmental harm. It considers both exergy losses and their environmental consequences, helping identify critical sources of impact^64^.

27 $$EIP=\frac{{\dot{Ex}}_{ls}}{{\dot{Ex}}_{in}}\times EDC$$

##### Environmental effect factor (EEF)

EEF compares the total environmental load to the useful exergy output. It provides insight into the overall ecological burden of the drying system. Lower EEF values indicate a more environmentally friendly and efficient drying process^64^.

28 $$EEF=\frac{{\dot{Ex}}_{ls}}{{\dot{Ex}}_{out}}$$

### Uncertainty analysis

The measurement uncertainties for temperature, relative humidity, wind speed, and solar radiation were found to be 0.34%, 0.29%, 0.23%, and 0.14%, respectively. Taking all these variables into account, the overall uncertainty in evaluating the efficiency of the PVT-ISD was estimated at around ± 2%.

29 $${\mathcal{W}}_{r}={\left[{\left(\frac{\partial R}{\partial {x}_{1}}{\mathcal{W}}_{1}\right)}^{2}+{\left(\frac{\partial R}{\partial {x}_{2}}{\mathcal{W}}_{2}\right)}^{2}+\dots +{\left(\frac{\partial R}{\partial {x}_{3}}{\mathcal{W}}_{3}\right)}^{2}\right]}^{1/2}$$

## Results and discussion

Throughout the experimental period, environmental conditions exhibited noticeable fluctuations, influencing the performance and thermal behavior of the photovoltaic-thermal integrated solar dryer (PVT-ISD). Ambient air temperatures measured in shaded locations ranged from 31 °C to 43 °C. Simultaneously, incident solar radiation showed considerable variation, fluctuating between 218 and 977 W/m^2^ over the course of the drying sessions. These changes in solar intensity played a crucial role in determining the thermal energy available for both the solar collector and the drying chamber.

In addition, natural wind speeds varied modestly, ranging between 0.1 and 0.4 m/s, which could have influenced the heat losses and air movement around the system. Within the PVT-ISD system, the temperature of the drying air was influenced by both the incident solar radiation and the selected airflow rate. At a higher airflow rate of 0.13 m^3^/s, the drying air temperature ranged from 35 °C to 65 °C, while at a lower airflow rate of 0.08 m^3^/s, the temperature range was slightly broader, from 34 °C to 70 °C. This variation reflects the combined effects of solar energy input, internal heat distribution, and airflow dynamics. The slightly higher maximum temperature at the lower airflow rate is attributed to the longer residence time of air within the dryer, allowing more heat absorption.

**Fig. 3:**
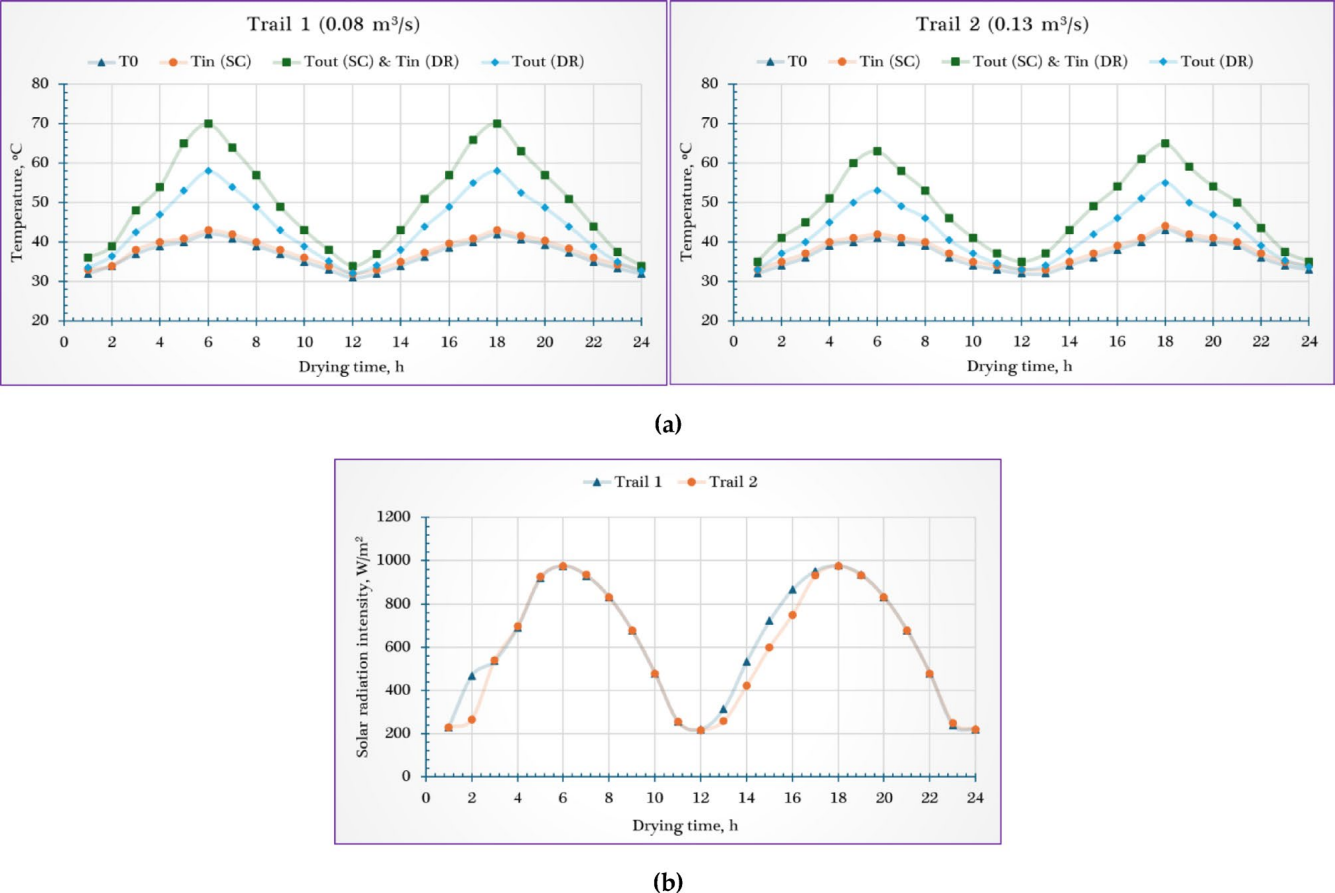
Weather conditions during field experiments. Whereas (**a**) temperature, and (**b**) solar radiation.

To better visualize and analyze these thermal behaviors, Fig. 3 provides detailed plots of the average solar radiation intensity and the corresponding temperature profiles recorded inside and outside the PVT-ISD during the drying trials conducted under both airflow conditions. These data offer insight into the system’s response to external environmental changes and help assess its thermal performance under variable operating scenarios.

### Drying kinetics

**Fig. 4:**
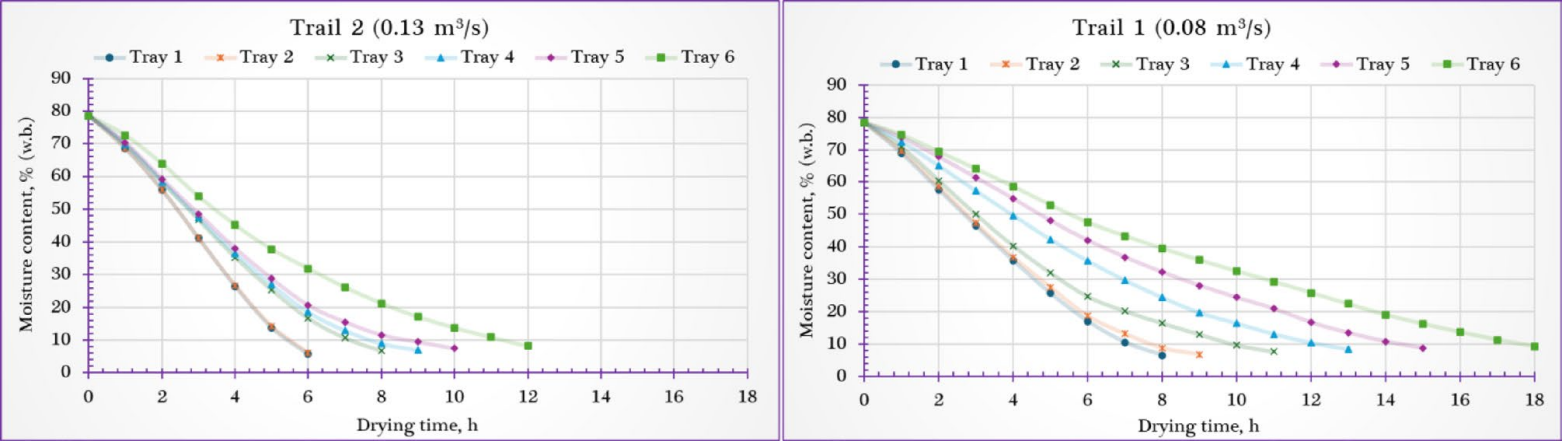
Moisture content of stevia leaf samples dried using the PVT-ISD.

Figure 4 illustrates the variation in MC of stevia leaves dried using the PVT-SD under different tray levels and airflow conditions. Roughly 750 g of fresh stevia leaves were evenly layered on each tray to a thickness of 3 cm. The initial MC was around 78.5% (w.b.). The time needed to reach the desired final moisture level varied between 6 and 18 h, depending largely on the tray’s vertical position and the airflow rate applied.

The fastest drying was observed on the lowest tray (tray 1), which is positioned nearest to the inlet of the heated air, especially when operated at the higher airflow rate of 0.13 m^3^/s. In contrast, the slowest drying occurred on the uppermost tray (tray 6), farthest from the air source and subjected to the lower airflow rate of 0.08 m^3^/s. The elevated temperature and stronger airflow near tray 1 enhanced the drying rate, achieving a final MC as low as 5.7%. On the other hand, samples dried on tray 6 at the lower airflow retained more moisture, with a final MC around 9.3%.

These findings are consistent with earlier studies^65–72^, which have shown that in multi-level or zoned drying systems, trays placed closer to the hot air inlet or in zones with more active airflow generally dry more quickly and reach lower final moisture levels than those positioned farther away^69,70,73^.

**Fig. 5:**
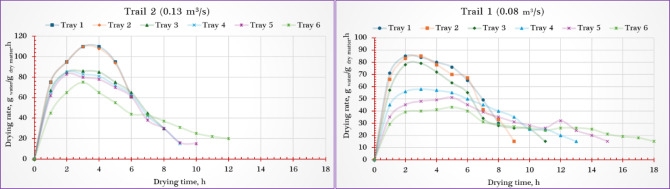
Drying rate of stevia leaf samples dried using the PVT-ISD.

As shown in Fig. 5, the data indicate a clear pattern: trays located in the lower sections of the PVT-ISD exhibited significantly faster drying rates than those positioned at higher levels. This can be attributed to the higher temperatures found near the air inlet, where hot air enters directly from the SC, enhancing both heat transfer and moisture removal.

For instance, when the PVT-ISD operated at an airflow of 0.13 m^3^/s, the drying rate on the lowest tray (tray 1) peaked at around 110 g _water_/g _dry matter_.h. In contrast, the uppermost tray (tray 6) showed a lower drying rate of approximately 75 g _water_/g _dry matter_.h. Additionally, increasing the airflow from 0.08 m^3^/s to 0.13 m^3^/s led to a notable increase in drying performance on tray 1—from roughly 85 to 110 g _water_/g _dry matter_.h —demonstrating the combined benefits of high airflow and optimal tray placement.

These observations are in line with earlier findings. Elshehawy et al.^44^ emphasized that higher drying air temperatures improve internal moisture diffusion to the surface, promoting faster evaporation. Likewise, Darvishi et al.^45^ noted that the drying process typically starts with a high rate when free moisture is readily available, followed by a gradual decrease as the product dries and internal moisture becomes harder to extract.

### Energy analysis of the SC

**Fig. 6:**
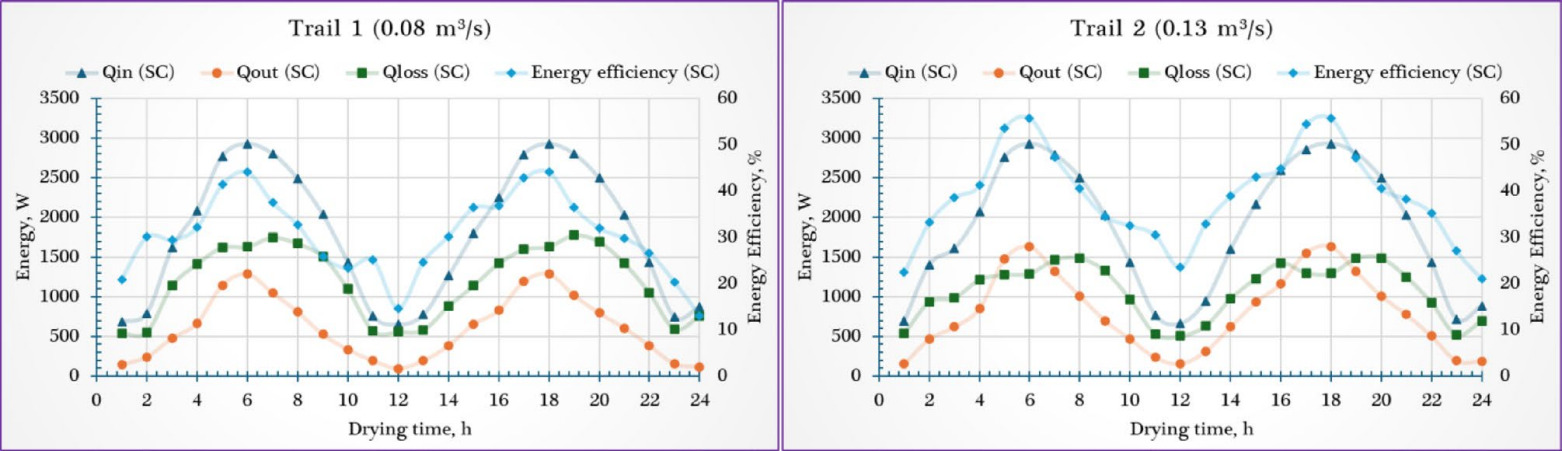
Energy analysis of the solar collector (${\dot{Q}}_{in, \mathrm{SC}}$, ${\dot{Q}}_{u, \mathrm{SC}}$, ${\dot{Q}}_{ls, \mathrm{SC}}$, and ${\eta }_{en, SC}$).

During the first trial at air flow rate of 0.08 m^3^/s, the ${\dot{Q}}_{in, SC}$ ranged from 654 to 2929 W throughout the day (Fig. 6). And the ${\dot{Q}}_{u, SC}$ varied between 95.73 W and 1292.4 W, largely influenced by the level of incident solar radiation. The ${\eta }_{e, SC}$ ranged between 13.03% and 44.14%. While during the second trial at air flow rate of 0.13 m^3^/s, the ${\dot{Q}}_{in, SC}$ ranged from 600 to 2931 W throughout the day (Fig. 6). And the ${\dot{Q}}_{u, SC}$ varied between 155.57 W and 1633.45 W, largely influenced by the level of incident solar radiation. The ${\eta }_{e, SC}$ ranged between 21.05% and 55.7%.

The ${\dot{Q}}_{u, SC}$ followed the trend of increasing with rising solar radiation, reaching its peak around midday for both airflow conditions. When comparing the two trials, the higher airflow rate of 0.13 m^3^/s resulted in a noticeable improvement in performance: the maximum ${\dot{Q}}_{u, SC}$ increased by approximately 26.4%, while the minimum value rose by about 62.5% compared to the lower airflow rate of 0.08 m^3^/s. Additionally, the ${\eta }_{e, SC}$ increased steadily during the morning hours, reaching its peak at 1:00 p.m. for both airflow rates, coinciding with the maximum solar intensity, and then gradually declined in the afternoon. When comparing the two airflow rates, the system operating at 0.13 m^3^/s demonstrated superior performance: its minimum efficiency was approximately 61.6% higher than that at 0.08 m^3^/s, while its maximum efficiency rose by about 26.2%, achieving a peak value of 55.7%.

The superior ${\dot{Q}}_{u, SC}$ and ${\eta }_{e, SC}$ observed at the higher airflow rate of 0.13 m^3^/s compared to 0.08 m^3^/s can be attributed to improved heat transfer dynamics within the PVT-ISD. At 0.13 m^3^/s, the increased air flow rate enhances convective heat transfer between the SC surface and the air flow, allowing more effective absorption and transport of thermal energy. Additionally, the higher mass flow rate results in greater energy transfer per unit time, even if the temperature rise is slightly lower. This condition also helps reduce the absorber plate’s temperature, minimizing thermal losses due to radiation and conduction. Furthermore, the more stable and uniform temperature distribution at the higher airflow rate contributes to more consistent energy utilization. In contrast, the lower airflow rate limits the volume of air passing through the PVT-ISD, reducing the total energy carried away and increasing ${\dot{Q}}_{ls, SC}$. Overall, the enhanced thermal exchange and reduced ${\dot{Q}}_{ls, SC}$ at 0.13 m^3^/s explain the improved performance in terms of both ${\dot{Q}}_{u, SC}$ and ${\eta }_{e, SC}$.

Several previous studies have demonstrated the significant impact of airflow rate on the ${\eta }_{e, SC}$. Machi et al.^74^ compared front and side entrance SCs, concluding that optimizing the inlet design—particularly with a front entrance—results in higher outlet temperatures and more effective heat removal from the absorber plate. The front entrance configuration achieved daily ${\eta }_{e, SC}$ of 37.2%, 40.4%, and 44.4% at increasing airflow rates, surpassing the side entrance by 3.1% to 3.7%. Similarly, Rani and Tripathy^75^ analyzed a flat plate SC and observed that increasing the air mass flow rate from 0.006 kg/s to 0.02 kg/s raised the ${\eta }_{e, SC}$ from 22.53% to 32.3%, while also significantly enhancing the convective heat transfer coefficient; they also noted that the influence of solar intensity on ${\dot{Q}}_{u, SC}$ and ${\eta }_{e, SC}$ was relatively minor. In another experimental study, Darici^76^ investigated a single-pass SC and found that higher mass flow rates increased ${\eta }_{e, SC}$ but led to lower outlet air temperatures, with the SC achieving a maximum outlet temperature of 66°C and an average ${\eta }_{e, SC}$ of 52%. Wang et al.^77^ introduced a flat-plate SC integrated with L-shaped dual micro heat pipe arrays and reported increasing ${\eta }_{e, SC}$ of 49.72%, 55.69%, 59.37%, and 61.48% corresponding to airflow rates of 80, 120, 160, and 200 m^3^/h, respectively, confirming a positive relationship between airflow rate and ${\eta }_{e, SC}$. Khelifa et al.^78^ conducted a numerical study on a PVT collector using both water and air as cooling mediums and found that increasing the number of fins and the intensity of solar radiation improved ${\eta }_{e, SC}$, although higher water flow rates caused a slight efficiency drop. Similarly, Dhaundiyal’s research on two-pass SCs with smooth and finned absorber plates showed that ${\eta }_{e, SC}$ consistently improved as airflow rate increased^79^.

### Exergy analysis

**Fig. 7:**
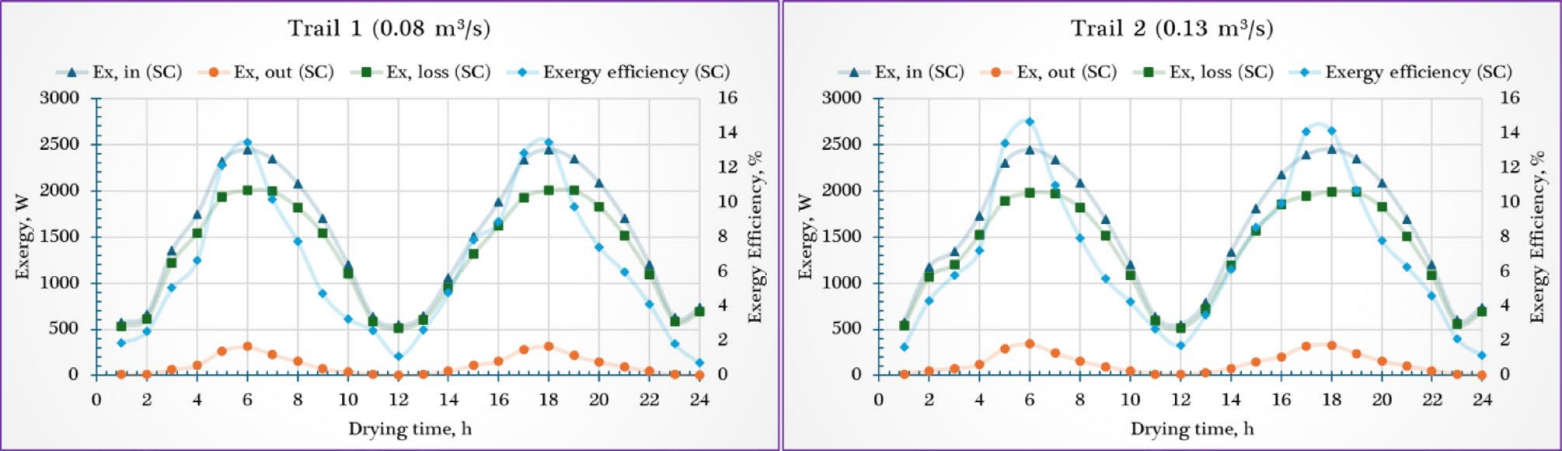
Exergy analysis of the SC (${\dot{Ex}}_{in, SC}$, ${\dot{Ex}}_{out, SC}$, ${\dot{Ex}}_{ls, SC}$ and ${\eta }_{ex, SC}$).

The exergy performance of the SC was assessed under two airflow conditions—0.08 m^3^/s and 0.13 m^3^/s—with notable differences in ${\dot{Ex}}_{in, SC}$ and ${\dot{Ex}}_{out, SC}$ flows as well as ${\eta }_{ex, SC}$ throughout the day (Fig. 7). For both trials, the ${\dot{Ex}}_{in, SC}$ followed a similar diurnal pattern, increasing in the morning with rising solar radiation, peaking around midday, and decreasing toward the afternoon. At 0.08 m^3^/s, ${\dot{Ex}}_{in, SC}$ ranged from 546.74 W to 2448.64 W, while at 0.13 m^3^/s, it ranged from 551.76 W to 2450.32 W, showing nearly identical maximum values and only a marginal (~ 0.9%) increase in the minimum value at the higher airflow rate. The ${\dot{Ex}}_{out, SC}$ exhibited greater improvement with airflow enhancement. At 0.08 m^3^/s, ${\dot{Ex}}_{out, SC}$ varied from 5.12 W to 312.74 W, whereas at 0.13 m^3^/s, it ranged between 8.08 W and 340.37 W. This reflects a 6.9% increase in maximum ${\dot{Ex}}_{out, SC}$ and a 57.8% increase in minimum ${\dot{Ex}}_{out, SC}$ when the airflow was raised, indicating more consistent and effective exergy delivery across all solar intensities. Regarding ${\eta }_{ex, SC}$, both trials showed the expected trend: rising during the morning, peaking at midday, and declining in the afternoon as solar input waned. However, the system operating at 0.13 m^3^/s demonstrated superior performance, with ${\eta }_{ex, SC}$ values ranging from 1.15% to 14.68%, compared to 0.73% to 13.49% at 0.08 m^3^/s. This represents a ~ 26% increase in minimum ${\eta }_{ex, SC}$ and a ~ 8.8% increase in maximum ${\eta }_{ex, SC}$ at the higher airflow rate.

**Fig. 8:**
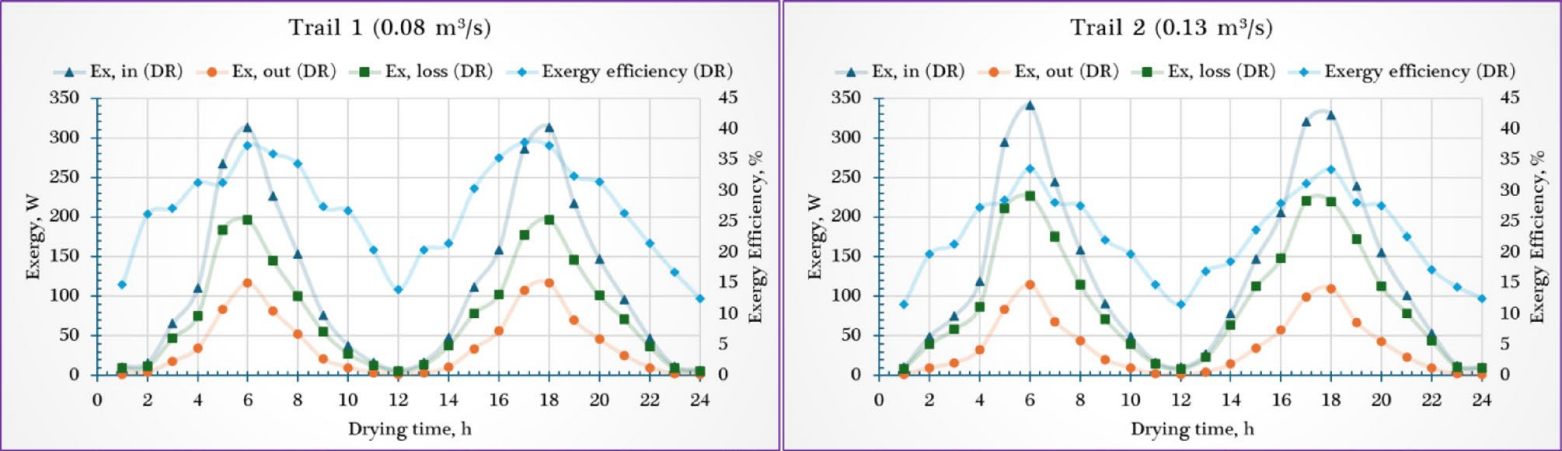
Exergy analysis of the DR (${\dot{Ex}}_{in, DR}$, ${\dot{Ex}}_{out,DR}$, ${\dot{Ex}}_{ls, DR}$ and ${\eta }_{ex, DR}$).

The exergy performance of the DR under two airflow conditions—0.08 m^3^/s and 0.13 m^3^/s—showed distinct differences in ${\dot{Ex}}_{in, DR}$ and ${\dot{Ex}}_{out, DR}$ as well as ${\eta }_{ex, DR}$ throughout the day (Fig. 8). In both cases, the ${\dot{Ex}}_{in, DR}$ followed a typical diurnal trend, increasing with solar radiation in the morning, peaking around midday, and decreasing in the afternoon. At 0.08 m^3^/s, ${\dot{Ex}}_{in, DR}$ ranged from 6.53 W to 313.3 W, while at 0.13 m^3^/s, it ranged from 10.30 W to 341.31 W, reflecting a 57.7% increase in minimum ${\dot{Ex}}_{in, DR}$ and a 9% rise in the maximum value with higher airflow. The ${\dot{Ex}}_{out, DR}$ also showed notable differences: it varied from 0.864 W to 116.96 W at 0.08 m^3^/s, and from 1.19 W to 114.69 W at 0.13 m^3^/s, indicating a 37.8% increase in the minimum output but a slight 1.9% decrease in the peak value. As for ${\eta }_{ex, DR}$, both airflow rates exhibited the same overall trend—rising to a peak at midday and declining later—but the lower airflow rate (0.08 m^3^/s) surprisingly outperformed the higher rate, with ${\eta }_{ex, DR}$ values ranging from 12.57% to 37.89%, compared to 11.56% to 33.6% at 0.13 m^3^/s.

The observed reduction in ${\eta }_{ex, DR}$ at higher airflow rates can be attributed to the imbalance between heat and mass transfer mechanisms. While an increased airflow velocity enhances the convective heat transfer coefficient and accelerates moisture removal, it also introduces greater irreversibilities within the DR. Specifically, excessive airflow results in higher energy consumption for moving air without a proportional increase in moisture evaporation, thereby lowering the overall ${\eta }_{ex, DR}$. Furthermore, at elevated velocities, the residence time of hot air in contact with the product is reduced, limiting effective energy utilization. These combined factors explain why, despite improved drying rates, exergy efficiency decreases as airflow velocity increases.

Several previous studies have highlighted the significant influence of air flow rate on the ${\eta }_{ex, SC}$. Motamed Sadr et al.^80^ evaluated a hybrid asphalt and façade-integrated SC and found that natural convection resulted in very low exergy efficiencies (0.13–0.22%) due to restricted airflow. However, when forced convection was introduced, performance improved markedly, with ${\eta }_{ex, SC}$ increasing to 0.94% at an airflow rate of 0.007 kg/s and reaching 1.67% at 0.014 kg/s. Similarly, Khalil et al.^81^ conducted experimental exergy analyses on single-flow SCs with different absorber plate geometries and showed that incorporating fins—particularly in inclined staggered arrangements—significantly enhanced exergy efficiency, with values reaching as high as 77%. Their findings also confirmed that ${\eta }_{ex, SC}$ increases with greater airflow rates. Supporting this trend, Ekka and Muthukumar studied a mixed-mode solar dryer equipped with double-pass SCs and reported that ${\eta }_{ex, SC}$ rose from 18.8% to 41.4% as the air mass flow rate increased from 0.018 kg/s to 0.062 kg/s. These studies collectively emphasize that both airflow enhancement and thoughtful SC design are critical for maximizing ${\eta }_{ex, SC}$ in solar thermal systems^56^.

### Sustainable indicators

To evaluate the thermodynamic efficiency and environmental sustainability of the PVT-ISD system, six key exergy-based indicators—Improved Potential (IP), Waste Exergy Ratio (WER), Sustainability Index (SI), Environmental Destruction Coefficient (EDC), Environmental Impact Factor (EIF), and Environmental Effect Factor (EEF)—were calculated for both the solar collector (SC) and drying room (DR) under airflow rates of 0.08 m^3^/s and 0.13 m^3^/s. Overall, the results revealed mixed trends across components and indicators depending on the airflow condition.

**Fig. 9:**
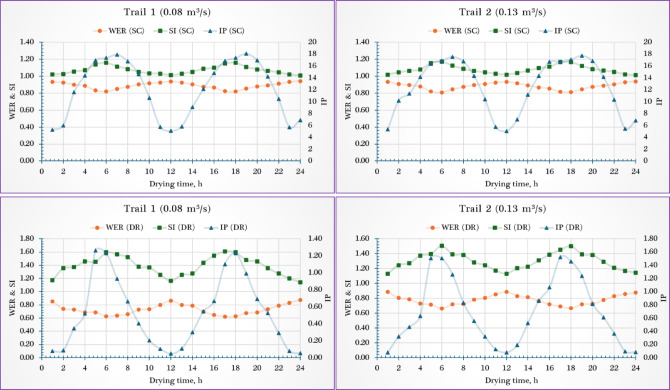
Sustainability indicators of the drying room (DR) and the solar collector (SC).

As shown in Fig. 9, the IP for the SC showed a slight increase of ~ 2.6% when airflow rose from 0.08 m^3^/s (12.11 W) to 0.13 m^3^/s (12.43 W), while the drying room (DR) experienced a ~ 5.3% decrease in IP (from 0.729 W to 0.69 W), indicating that higher airflow enhances energy recovery in the solar collector (SC) but slightly reduces potential in the DR due to reduced residence time. The WER decreased marginally in the SC from 0.888 to 0.881 (~ 0.8% reduction), indicating slightly lower waste under higher airflow. Conversely, WER in the DR increased from 0.729 to 0.775 (~ 6.3% increase), suggesting more exergy losses likely due to less heat absorption in faster-moving air. For the SI, the SC showed a minor improvement from 1.069 to 1.078 (~ 0.84% increase), while the DR saw a ~ 5.8% decrease, dropping from 1.38 to 1.30. These patterns suggest that higher airflow improves overall system sustainability in the SC but slightly weakens it in the DR.

The observed IP values align with the previous studies 12.75 W^55^, and 17 W^82^. While Eqs. (25 and 26) were used to compute both WER and SI. Where the WER ranged between 1.15 to 1.36, this value was slightly higher than previous studies, 0.41 and 0.445^55^, and 0.38 and 0.55^82^. Moreover, the SI value ranged from 1.09 to 1.28. These results lie in the range 1.26 to 1.71^56^, 1.12 to 2.57^83^, and 1.30^84^.

### Environmental indicators

**Fig. 10:**
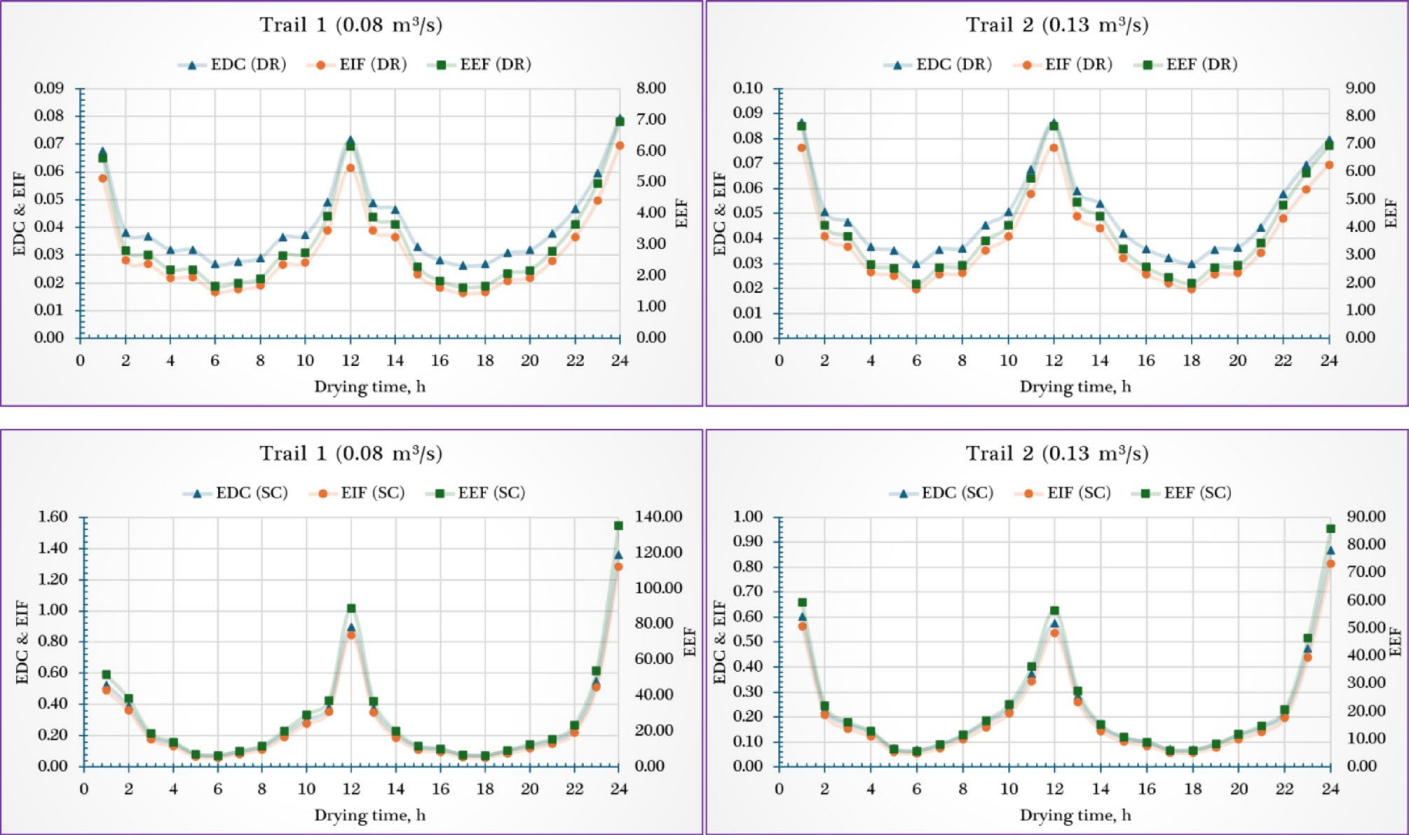
Environmental indicators of the drying room (DR) and the solar collector (SC).

As shown in Fig. 10, the EDC dropped in the SC from 0.291 to 0.234 (~ 19.6% reduction), indicating significantly reduced thermodynamic degradation at higher airflow. However, the DR’s EDC increased from 0.041 to 0.049 (~ 19.5% increase), suggesting marginally higher exergy-related environmental burden. Similarly, the EIF declined in the SC by ~ 20.6% (from 0.267 to 0.212), reflecting better environmental performance with increased airflow. In contrast, the DR experienced a ~ 25.8% rise (from 0.031 to 0.039), pointing to a slight environmental drawback under faster airflow. Finally, the EEF showed a notable ~ 20.4% decrease in the SC (from 28.13 to 22.38), reinforcing improved environmental compatibility at 0.13 m^3^/s. However, in the DR, EEF rose significantly by ~ 27.4% (from 3.089 to 3.935), aligning with the observed increases in EDC and EIF.

Kushwah et al.^64^ evaluated the exergetic-based sustainability indicators of an advanced heat exchanger-evacuated tube assisted drying system. Indicators such as ESI, EDC, and EEF were assessed using garlic cloves, banana slices, and peppermint leaves. The drying cabin reached 87 °C in Case-I and 61 °C in Case-II. Case-I showed higher sustainability with an ESI of 2.92, while Case-II had a lower value of 1.25, reflecting poor energy utilization. Results suggest that better absorption of biomass-generated energy and recovery of waste heat can enhance efficiency. EEF and EDC values ranged from 0.98–15.36 and 1.47–16.43, respectively.

## Conclusions

This experimental study evaluated the drying performance of *Stevia rebaudiana* leaves in a photovoltaic thermal–indirect solar dryer (PVT-ISD), focusing on the combined effects of tray position and airflow rate on drying kinetics, energy–exergy performance, and sustainability.

The results clearly demonstrated that both airflow rate and tray position significantly influence drying behavior. Leaves placed on the lowest tray, closest to the hot air inlet, achieved the fastest drying (6 h at 0.13 m^3^/s) with a final moisture content of 5.7%, while those on the uppermost tray required up to 18 h (0.08 m^3^/s), retaining 9.3% moisture. These findings underscore the need to optimize tray arrangement and airflow distribution to achieve uniform drying.

From an energy and exergy perspective, increasing the airflow rate to 0.13 m^3^/s substantially improved solar collector performance, raising thermal efficiency by over 26% and exergy efficiency by up to 8.8%. However, the drying chamber performed better at the lower airflow rate, benefiting from longer residence time and more effective heat absorption. This highlights a trade-off between maximizing collector efficiency and achieving optimal drying conditions inside the chamber.

Sustainability and environmental indicators reinforced these observations. Higher airflow improved solar collector sustainability and reduced environmental impact, but slightly compromised the drying chamber’s performance. Such results suggest that careful balance in airflow design is critical for minimizing environmental burden while enhancing dryer efficiency.

Overall, this study demonstrates that optimizing airflow and tray configuration is essential not only for energy and exergy efficiency but also for ensuring environmentally sustainable drying. These insights provide valuable guidance for the practical design of solar dryers to support sustainable agriculture and the large-scale drying of stevia leaves.

Future studies should focus on optimizing airflow distribution using design modifications or CFD-based modeling to improve drying uniformity across trays. Integrating intelligent control systems and thermal storage could stabilize operating conditions and enhance energy efficiency. Environmental sustainability can be further assessed through life cycle analysis and renewable–hybrid configurations, while linking energy performance with product quality will provide more holistic evaluation. Finally, scale-up and techno-economic studies are recommended to support industrial application of PVT–ISD systems in stevia processing.

## Data Availability

All data are presented within the article.

## List of symbols

$$MC$$: Moisture content
$$W$$: Sample weight
${\dot{m}}_{a}$: Mass flow rate
${\dot{E}}_{a}$: Energy flow rate
$${h}_{a}$$: Enthalpy
$${v}_{a}$$: Air velocity
$${z}_{a}$$: Height
$$g$$: Gravity acceleration
$$\dot{W}$$: Work done
$$\dot{Q}$$: Heat transfer
$${\dot{Q}}_{u}$$: Useful energy
$${\dot{Q}}_{in}$$: Input energy
$${\dot{Q}}_{ls}$$: Energy loss
${I}_{s}$: Solar radiation intensity
${A}_{SAC}$: Surface area of the solar collector
${C}_{pa}$: Specific heat of air
$${T}_{c}$$: Air temperature
$${\eta }_{en}$$: Energy efficiency
${m}_{w}$: Quantity of removed water from date sample
$L$: Latent heat of vaporization of water
$${t}_{d}$$: Drying time
$${\eta }_{ex}$$: Exergy efficiency
$$u$$: Internal energy
$$s$$: Entropy
$\alpha$: Absorptivity of glass
$$\dot{Ex}$$: Exergy
$\tau$: Transmissivity of glass
$${\mu }_{ch}$$: Chemical energy
$${T}_{0}$$: Atmospheric temperature

## Subscription

$$i$$: Inlet
$$o$$: Outlet
$$\mathrm{SC}$$: Solar collector
Dryer: The solar dryer
DR: Drying room

## Abbreviations

PVT-ISD: Photovoltaic thermal–indirect solar dryer
EDC: Environmental destruction coefficient
EIF: Environmental impact factor
DR: Drying Room
EEF: Environmental effect factor
OAD: Open air drying
IP: Improvement Potential
WER: Waste Exergy Ratio
SI: Sustainability Index
SD: Solar Dryer
GHG: Greenhouse gas
PV: Photovoltaic
MC: Moisture content
